# Microbial inoculums improve growth and health of *Heteropneustes fossilis *via biofloc-driven aquaculture

**DOI:** 10.1186/s12934-023-02107-0

**Published:** 2023-06-02

**Authors:** Vikash Kumar, Himanshu Sekhar Swain, Paton Vuong, Suvra Roy, Aurobinda Upadhyay, Ramesh Chandra Malick, Kampan Bisai, Parwinder Kaur, Basanta Kumar Das

**Affiliations:** 1 Aquatic Environmental Biotechnology and Nanotechnology (AEBN) Division, ICAR-Central Inland Fisheries Research Institute (CIFRI), Barrackpore, 700120 India; 2 Fisheries Resource Assessment and Informatics (FRAI) Division, ICAR-Central Inland Fisheries Research Institute (CIFRI), Barrackpore, 700120 India; 3 ICAR-Central Institute of Freshwater Aquaculture, Kausalyaganga, 751002 India; 4 UWA School of Agriculture and Environment, The University of Western Australia, Perth, 6009 Australia; 5 ICAR-Central Inland Fisheries Research Institute (CIFRI), Barrackpore, 700120 India

**Keywords:** Aquaculture, Biofloc technology, Microbial inoculums, Water quality, Growth performance, Immunity, *Heteropneustes fossilis*

## Abstract

Biofloc technology aims to maximize fish farming productivity by effectively breaking down ammonia and nitrite, promoting healthy flocculation, and enhancing the growth and immunity of cultured animals. However, a major limitation in this field is the suitable starter microbial culture and narrow number of fish species that have been tested with the biofloc system. Here, we investigated various microbial inoculum containing beneficial microbes with probiotics, immunostimulatory and flocs development and bioremediation properties would lead to the development of ideal biofloc development. Three treatment groups with different microbial combinations, viz., group 1 [*Bacillus subtilis* (AN1) + *Pseudomonas putida* (PB3) + *Saccharomyces cerevisiae* (ATCC-2601)], group 2 [*B. subtilis* (AN2) + *P. fluorescens* (PC3) + *S. cerevisiae* (ATCC-2601)] and group 3 [*B. subtilis* (AN3) + *P. aeruginosa* (PA2) + *S. cerevisiae* (ATCC-2601)] were used and compared with the positive control (pond water without microbial inoculums) and negative control (clear water: without microbial inoculums and carbon sources) on biofloc development and its characteristic features to improve the water quality and growth of fish. We demonstrated that microbial inoculums, especially group 2, significantly improve the water quality and microbiota of flocs and gut of the test animal, *Heteropneustes fossilis*. The study further demonstrates that biofloc system supplemented with microbial inoculums positively regulates gut histomorphology and growth performance, as evidenced by improved villous morphology, amylase, protease and lipase activity, weight gain, FCR, T3, T4 and IGF1 levels. The inoculums induced an antioxidative response marked by significantly higher values of catalase (CAT) and superoxide dismutase (SOD) activity. Furthermore, the supplementation of microbial inoculums enhances both specific and non-specific immune responses and significantly elevated levels of immune genes (transferrin, interleukin-1β and C3), and IgM was recorded. This study provides a proof-of-concept approach for assessing microbial inoculums on fish species that can be further utilized to develop biofloc technology for use in sustainable aquaculture.

**Supplementary Information:**

The online version contains supplementary material available at 10.1186/s12934-023-02107-0.

## Introduction

Climate change brings a cascade of threats to agroecosystems, affecting production and global food security [1–3]. These deleterious effects are observed in the physical condition and physiology of farmed aquatic animals and ecosystem structures, along with disruptions to supplies and product prices (cost of fish oil and fish meal), the primary and secondary productivity of water bodies, as well as other goods and services required for sustainable production. Aquaculture plays a substantial role in sustainable food production and is responsible for feeding roughly 800 million people, [4-8] with significant involvement in nutritional security for meeting current and future demands towards the provision of quality animal protein [9, 10]. Despite advances in aquaculture practices, several developing countries are still not self-sufficient in this sector. Common problems in aquaculture production include prolonged droughts due to climate change, the (re)-emergence of virulent pathogens, issues from leftover feed and metabolic waste product accumulation, deficient oxygen levels, wounds and injuries resulting from animal-to-animal interactions, and supply issues in fishmeal procurement for use in fish feed [2, 8, 11–14]. Together these can generate environmental stress conditions which affect the growth, immunity and survival of farmed aquatic animals [15, 16].

In Southeast Asian countries, stinging catfish (*Heteropneustes fossilis*) is a well-known indigenous farmed catfish species, having several health benefiting properties [17]. With high demand, intensification and commercialisation of indigenous catfish aquaculture, farmers are frequently facing disease outbreaks and heavy mortalities due to microbial infections [18]. In case of *H. fossilis*, the proliferation of pathogenic and opportunistic microorganisms lead to decreased growth and food utilisation, and in many instances, massive mortality rates [19]. Hence, improved culture technology and better management of the farmed environment could improve the immunity of the stinging catfish and generate resistance against pathogenic microbes.

Biofloc technology offers a sustainable pollution-free and cost-effective cultivation approach by improving water quality through the production of microbial proteins within the aquatic agroecosystem, subsequently enhancing the growth, health and survival of cultivated animals [20, 21]. This technology is considered a new blue revolution in the aquaculture sector which will not only meet the growing demand for quality animal protein, but also tackle major environmental, water scarcity, and animal health/disease issues [22, 23]. The basic principle of biofloc technology is to transform and recycle excessive nutrients and waste, such as inorganic nitrogen products (e.g., NH_3_-N and NO_2_-N) generated from uneaten feed and faeces, and convert them into microbial biomass. This process is mainly carried out by heterotrophic bacteria, which also utilizes carbohydrate content from uneaten feed and faeces, along with other external carbon sources to help regulate the carbon/nitrogen ratios within the system [24–29]. The bacterial biomass or bioflocs contain high protein content, and the *in-situ* utilization through feed supplements of this bacterial protein source imparts beneficial effects to the farmed animals. Additionally, the microbe-associated molecular pattern (MAMP) and microbially bioactive components such as vitamins, carotenoids, antioxidants, minerals and glutathione present in flocs are reported to help nutritionally modulate immune response, resulting in improved growth performance and disease resistance against pathogenic microbial infections in farmed fish [28, 30, 31].

The sustainability of the biofloc system is linked to both the diversity of the microbiota and the farmed species. There is limited knowledge regarding the cultivation of fish species aside from shrimp, Nile tilapia and *Pangasius* sp. using biofloc treatment as a sole aquaculture production system or in combination with other production systems [23, 32, 33]. Biofloc technology is regulated by the biofloc microbiome, which helps to maintain water quality within the system and improves feed utilization by the cultivated animals [34, 35]. The choice of microbial inoculums, also known as the starter cultures, are believed to have a significant role in biofloc development, floc and host-microbiome characteristics, as well as drive improvements in growth performance and immune response of cultivated species [36, 37].

In this study, we aimed to build a standard biofloc development protocol by investigating various microbial inoculums to gauge the development within the resultant systems in whether they enabled the efficient use of water and nutrients, provided and maintained optimum water quality, as well as improved the gut histomorphology and growth performance of *H. fossilis*. In addition, we investigated whether the microbial inoculums contributed to the maintenance of the beneficial microbiome in both the floc and host*.* We also aimed to unravel how the addition of microbial inoculums results in the generation of antioxidant and protective immune responses in *H. fossilis*.

## Materials and methods

### Experimental setup

The study was performed in the biofloc units of ICAR-Central Inland Fisheries Research Institute, Kolkata, India. In the experimental culture units, fifteen fibre-reinforced plastic (FRP) tanks (1000 l capacity) were used, each filled with 750 l of freshwater. Photoperiods of 12 h light and 12 h darkness and optimum water temperatures between 27.5 and 28.5 °C were maintained throughout the whole study. A single batch of juvenile Asian stinging catfish (*H. fossilis*) were obtained from the local fish seed market and was acclimatized to the experimental conditions for 1 week with the initial average body length being 4.9 ± 0.061 cm and weight 1.72 ± 0.022 g. The fish were distributed randomly in the tanks at a density of 600 numbers in each tank (800 catfish m^−2^) and fed with a commercial floating catfish diet (Growel Feeds Pvt. Ltd., India, 35% crude protein and 10% crude fat) at 5% of the body weight twice a day (at 9.00 h and 16.00 h) for 91 days. Based on the fish biomass estimates the feed rations were adjusted every week. Chemicals, antibiotics and other medicines were strictly avoided during the experiment period.

Three treatment groups using inoculations of different microbial combinations, viz., group 1 [*Bacillus subtilis* (AN1) + *Pseudomonas putida* (PB3) + *Saccharomyces cerevisiae* (ATCC-2601)], group 2 [*B. subtilis* (AN2) + *P. fluorescens* (PC3) + *S. cerevisiae* (ATCC-2601)] and group 3 (*B. subtilis* (AN3) + *P. aeruginosa* (PA2) + *S. cerevisiae* (ATCC-2601), along with a baseline control (pond water without microbial inoculums) and negative control (clear water: without microbial inoculums and addition of carbon sources) were used, and each were evaluated in triplicate (Tables 1, 2). Approximately 20% of water was exchanged daily within the negative control group, whereas in the treatment groups water was added in regular intervals to make up for water loss due to evaporation. For biofloc development in the treatment and baseline control group, initially the culture water was inoculated with pond water and subsequently, a carbon source (jaggery) was added daily at an estimated C/N ratio of 15:1, two hours after feeding (Additional file 1: Fig. S1) [38, 40].

**Table 1:** Microbial inoculums used in the preparation of flocs

Bacteria species	Properties
1. *Bacillus subtilis* (AN1)	Probiotic
2.* Bacillus subtilis* (AN2)	
3.* Bacillus subtilis* (AN3)	
4.* Pseudomonas putida* (PB3)	Floc formation and bioremediation properties
5.* Pseudomonas fluorescens* (PC3)	
6.* Pseudomonas aeruginosa* (PA2)	

*Yeast species*	Properties
1.* Saccharomyces cerevisiae* (ATCC-2601)

**Table 2:** Experimental design of the study

Inputs	Negative control	Baseline control	Treatment groups		
			Group 1	Group 2	Group 3
Pond water	–	3 l	3 l	3 l	3 l
C/N ratio	–	15	15	15	15
Carbon source	–	jaggery	jaggery	jaggery	jaggery
Nitrogen source	Feed (10%)	Ammonium sulphate + feed (5%)	Ammonium sulphate + feed (5%)	Ammonium sulphate + feed (5%)	Ammonium sulphate + feed (5%)
Microbial inoculum (1:1:1)	–	–	*B. subtilis* (AN1) + *P. putida* (PB3) + *S. cerevisiae* (ATCC-2601)	*B. subtilis* (AN2) + *P. fluorescens* (PC3) + *S. cerevisiae* (ATCC-2601)	*B. subtilis* (AN3) + *P. aeruginosa* (PA2) + *S. cerevisiae* (ATCC-2601)
Stocking density (800 fish m^−3^)	600 nos./tank	600 nos./tank	600 nos./tank	600 nos./tank	600 nos./tank

### Water quality

Temperature, dissolved oxygen, salinity and pH were measured daily using a portable photometer multiparameter (Aquaread AP 7000, UK), Refractometer (VWR, India) and pH meter (Eutech, India). Inorganic dissolved nitrogen including NO_3_^−^-N (nitrate nitrogen) and NO_2_^−^-N (nitrite nitrogen) were measured in every 2 days interval using a volumetric analysis method following a standard protocol (APHA 2017) [39]. The total biofloc volume was determined following 15–20 min of sedimentation of biofloc water in Imhoff cones. To monitor the total suspended solids (TSS), the biofloc water samples were filtered every second day using 0.6-μm glass fiber micro-filters (GF-6, Macherey-Nagel, Düren, India) [41]. The samples were processed in a muffle furnace and weight difference of the dried samples before and after the processing was used for the determination of volatile suspended solids (VSS). Total ammonia nitrogen (TAN) (NH_4_^+^-N + NH_3_) and Kjeldahl nitrogen (Kj-N) were analysed using standard methods [42]. The difference between TAN and Kj-N were used to determine the biofloc protein content by multiplying a 6.25 conversion factor with organic nitrogen content [25, 43].

### Bacterial enumeration from biofloc and gut samples of *H. fossilis*

Organization for Economic Cooperation and Development (OECD) guidelines were followed for the handling and care of experimental animals. The animal utilization protocol was approved by Institutional Animal Ethics Committee, ICAR-Central Inland Fisheries Research Institute, Kolkata, India, (IAEC/2021/04) for the experimental setup.

The biofloc samples were used to determine the abundance of total cultivable bacteria following the protocol developed by Guan et al. [44] with slight modifications. In brief, the samples of floc (10 ml culture water) were dispersed into 90 ml of sterilized distilled water and incubated for 60 min at 28 °C with shaking at 120 rpm [44]. The resulting slurry was then diluted serially with sterilized solution of physiological saline (0.9%, w/v). For the isolation of bacteria, aliquots from each serial dilution (0.1 ml) were spread onto Petri dishes containing Tryptone soya agar (TSA) media and incubated overnight at 28 °C. To avoid the growth of fungal contaminants, nystatin (50 mg L^−1^) were supplemented in the solidified media. The plates consisting of 30–300 CFU ml^−1^ at particular dilutions were used to count the number of colonies and calculate the abundance of bacteria in each biofloc sample.

For bacterial enumeration from *H. fossilis* gut samples, the fish were randomly selected from different treatment and control groups, anesthetized with clove oil (50 µl l^−1^ water), stored in sterile plastic bags at 4 °C and were processed within 24 h of collection. The surface of *H. fossilis* was disinfected with 70% alcohol, dissected under aseptic conditions and the intestines were taken out and cut into small pieces. Tissue samples were homogenized aseptically in 10 ml distilled water for 15–30 s at room temperature using a tissue homogenizer (Borg Scientific, India). Similarly, the homogenate was diluted serially with sterilized physiological saline solution and aliquots of each dilution (0.1 ml) were spread onto Petri dishes containing TSA media and overnight incubated at 28 °C with shaking at 120 rpm. The plates consisting of 30–300 CFU ml^−1^ at particular dilutions were used to count the numbers of colonies and calculate the abundance of bacteria in each fish gut sample group.

### Enumeration of *Bacillus* and *Pseudomonas *from biofloc and gut samples of *H. fossilis*

Selective media was used to enumerate the *Bacillus* and *Pseudomonas* from biofloc and gut samples of *H fossilis*. The process followed the aforementioned bacterial enumeration methods, whereby the floc samples (10 ml culture water) were dispersed into 90 ml of sterilized distilled water and incubated at 28 °C for 60 min. After serial dilution in sterilized physiological saline solution (0.9%, w/v), the 0.1 ml of aliquots from each dilution were spread onto Petri dishes containing either HiCrome™ *Bacillus* Agar (HiMedia, India) or *Pseudomonas* Agar Base (HiMedia, India) and incubated overnight at 28 °C with shaking. For growth suppression of fungal contaminants, the solidified media were supplemented with nystatin (50 mg l^−1^). The plates consisting of 30–300 CFU ml^−1^ at particular dilutions were used to count the number of colonies and calculate the abundance of *Bacillus* and *Pseudomonas* in each biofloc sample.

Fish were again randomly selected from different treatment and control groups, stored in sterile plastic bags at 4 °C and were processed for *Bacillus* and *Pseudomonas* enumeration in the same manner as the previous bacterial enumeration. To reiterate, the surface of *H. fossilis* was disinfected and dissected and the intestines were taken out and cut into small pieces. The tissue sample was homogenized aseptically in 10 ml distilled water and the homogenate was serially diluted with sterilized physiological saline solution. The aliquots (0.1 ml) of each dilution were spread onto Petri dishes containing either HiCrome™ *Bacillus* Agar (HiMedia, India) or *Pseudomonas* Agar Base (HiMedia, India) and incubated overnight at 28 °C with shaking. The plates consisting of 30–300 CFU ml^−1^ at particular dilutions were used to count the numbers of colonies and calculate the abundance of bacteria in fish gut samples from each experimental group.

### Growth performance

To calculate the growth performance and survival of *H. fossilis*, 30 fish were randomly selected from treatment and control groups post experiment. Feeding was withheld before sampling. The parameters such as specific growth rate (%), weight gain (%), weekly weight gain (g wk^−1^) (WG) and FCR were determined as follows:

$$\begin{aligned} & {\text{SGR}}\,\left( \% \right) = 100 \times \left( {{\text{ln average final weight}}\,\left( {\text{g}} \right) - {\text{ln average initial weight}}\,\left( {\text{g}} \right)} \right){\text{/time}}\,\left( {\text{culture day}} \right) \\ & {\text{Weight gain}}\,\left( \% \right) = 100 \times \left( {{\text{Final weight}}\,\left( {\text{g}} \right) - {\text{Initial weight}}\,\left( {\text{g}} \right)} \right)/{\text{Initial weight}} \\ & {\text{Weekly weight gain}}\,\left( {{\text{g wk}}^{ - 1} } \right) = \left( {{\text{Final weight}}\,\left( {\text{g}} \right) - {\text{Initial weight}}\,\left( {\text{g}} \right)} \right)/{\text{time}}\,\left( {{\text{week}}} \right) \\ & {\text{FCR}} = {\text{Total dry feed intake}}\,\left( {\text{g}} \right)/{\text{weight gain}}\,\left( {\text{g}} \right). \\ \end{aligned}$$

### Sample collection for biochemical analysis

Five fish were randomly selected from each control and treatment group to collect serum and tissue samples for biochemical analysis. In brief, the fish were anesthetized with clove oil (50 µl l^−1^ water), and blood and tissue samples including gill, muscle, kidney, liver and gut were collected. Sterile conditions were maintained during all the collection procedures. Blood samples were collected first using a 2 ml hypodermal syringe by puncturing the caudal vein of the fish. The blood samples were collected without anticoagulant in sterile Eppendorf tubes, and were stored overnight at 4 °C. Later, the blood samples were centrifuged at 4000 × *g* at 4 °C for 10 min and the straw-coloured serum samples were collected and stored at − 20 °C until further analysis. The collected tissue samples were homogenized using tissue lyser (Qiagen, Hilden, Germany), and centrifuged at 4 °C for 10 min at 10,000 rpm and the collected supernatant was stored at − 80 °C.

### Digestive enzyme assay

At the end of the experimental period, fish from each control and treatment group were dissected and gut tissue samples were kept in 0.25 M sucrose solutions. Amylase activity was analysed using the 3,5-dinitrosalicylic acid method by estimating the reducing sugar production by α-amylase and glucoamylase. The lipase activity was analysed based on a titration method using a phenolphthalein indicator [45, 46]. The digestion method of casein (pH 7.8, triphosphate buffer and trichloroacetic acid) was also performed to estimate the levels of protease activity in the gut samples [47].

### Antioxidant enzymes assay

The activity of metabolic and antioxidant enzymes was measured in the kidney and gill tissue following standard protocols. The activity of superoxide dismutase (SOD) was analysed in a medium containing sodium carbonate buffer (pH 10.2), EDTA, epinephrine and enzyme extract [48]. The differences in absorbance were observed in a Microplate reader (BioTek Epoch^TM^2 Plate Reader, USA) at 480 nm. The Calibrone [49] method was used to analyse catalase (CAT) activity. Briefly, the intensity of H_2_O_2_ breakdown was determined by measuring the assay absorbance at 240 nm. The solution mixture comprised of 7.2 pH 50 mM phosphate buffer and 50nM of H_2_O_2_. The solution was calibrated in Microplate Reader (BioTek Epoch^TM^2 Plate Reader, USA) to 320 nm H_2_O_2_ having a coefficient extinction of 40 M^−1^ cm^−1^. The activity of CAT is expressed as one unit of H_2_O_2_ decomposed per milligram of protein per min.

### Serum biochemical indices and immune-stress responses

Total protein in fish serum obtained from control and biofloc treatment groups were measured using an automated biochemical analyzer (Transasia Erba EM–200, Auto Analyzer, USA) after the experimental period. The analysis was performed in two independent experiments, in triplicate for each analysis. The tri-iodothyronine (T3) and thyroxine (T4) in the serum of fish were analyzed using an ELISA (enzyme-linked immune survey assay) kit (BT Bioassay, Shanghai, China) following the manufacturer’s instructions. The final OD value was measured at 450 nm using a Microplate reader.

A commercial ELISA kit obtained from the Bioassay technology laboratory, China, was used for the analysis of cortisol according to the manufacturer's protocol. From the standard solution of cortisol, i.e., 0, 20, 50, 100, 200, 400 and 800 ng ml^−1^, 20 μl of each solution along with fish serum samples were added to the microplate in triplicate. Simultaneously, 200 μl of horseradish peroxidase enzyme conjugate was added to each well. The wells were mixed gently for 10 min and incubated at room temperature for 1 h. Later, each well solution was removed by washing the plate with PBS 400 μl three times and shaking out the content onto absorbent paper to remove residual drops that could affect the precision and accuracy of the assay. Subsequently, 100 μl of tetramethyl benzidine (TMB) enzyme substrate was added to all wells and incubated at room temperature for 15 min. The reaction of enzymes was observed by a change in the color, which was stopped by adding 100 μl of 0.5 M phosphoric acid (H_2_PO_3_). The color intensity was inversely proportional to the cortisol concentration in the samples. Afterward, a microtiter plate reader (spectrophotometer) was used to measure the absorbance at 450 nm within 10 min of the addition of the stop solution.

To quanitfy immunoglobulin M (IgM), a commercial ELISA kit obtained from Bioassay technology laboratory, China, was used to measure the IgM activity in serum samples of fish following the manufacturer's protocol. Briefly, 50 μl of IgM standard solution were added to biotinylated antibody containing standard wells. Later, 10 μl of anti-COR antibody, 40 μl of serum sample and 50 μl of streptavidin-HRP were added into the microplate wells. The solution in the plate was thoroughly mixed, covered with sealer and incubated at 37 °C for 60 min. Afterwards, the plate sealer was removed and washed 5 times with aprroximately 350 μl of wash buffer, allowing the wash buffer to sit for 30 s to 1 min between each wash. 50 μl each of substrate solution A and B were added into each well, sealed and incubated in dark conditions at 37 °C for 10 min. Later, 50 μl of stop solution was added into each well and a color change from blue to yellow color was observed. Within 10 min after the addition of the stop solution, the OD was measured at 450 nm in a microplate reader. Similarly, IGF1 (insulin-like growth factor 1) was quantified in fish serum by an ELISA (enzyme-linked immune survey assay) kit obtained from BT Bioassay, Shanghai, China. Following the manufacturer’s protocol, the assay was performed and final OD value was taken in microplate reader at 450 nm. All assay kits were previously used for the biochemical indices and immune-stress responses analysis of serum in fish models [18, 50].

### Histological analysis

The fish cultured in different treatment and control groups (~ 5 nos.) were anesthetized with clove oil (50 µl l^−1^ water) and gut tissue were collected at the end of the experiment. Investigation on any possible gross lesions in internal organs were done and recorded during the post-mortem examination. The collected gut tissue samples were first fixed in 10% NBF (neutral buffered formalin). Later, the fixed tissues were washed and cut into small pieces roughly 1–2 mm in size. Using different gradients of ethanol, the samples were dehydrated and treated with xylene (clearing agent). Using an impregnation technique, the processed tissues were embedded into paraffin using the Leica EG 1140H embedding machine, Germany. The paraffin-embedded tissue was sectioned, maintaining a 5 μm thickness, with a microtome and stained with Hematoxylin and Eosin [50, 51]. Later, the processed sections were visualized for cellular changes under a light microscope.

### RNA extraction and reverse transcription

The total RNA was isolated with Trizol^®^ reagent using the manufacturer’s standard protocol. Briefly, 3 individual fish per experimental group after 35, 70 and 91 days were washed with sterile freshwater, dissected, immediately frozen in liquid nitrogen and stored at − 80 °C. The sample tissue was aseptically homogenized for 15–30 s with 1 ml chilled Trizol^®^ at room temperature and incubated at 20 °C for 5 min. After this step, 200 μl of chloroform was added to the homogenate, and mixed for 15 min vigorously at 20 °C, then centrifuged for 10 min at 10,000 rpm. The aqueous upper layer was collected in a new tube with an addition of 500 μl of isopropanol. The solution was then kept for 2 h at − 20 °C and again centrifuged for 10 min at 10,000 rpm. The obtained pellet was washed using 75% ethanol, centrifuged for 10 min at 7,000 rpm and briefly air-dried to remove any traces of ethanol. Following this, 50 μl of DEPC-treated sterile water was used to dissolve the RNA pellets and with the suspension stored at − 20 °C until further analysis. To remove contamination of genomic DNA, the RNA samples were treated with RNase free DNAse I (Thermo Scientific, India). To check the quality and concentration (ng µl^−1^) of isolated RNA, the absorbance was measured in the NanoDrop Spectrophotometer (Thermo Scientific, India) at 260/280. Afterward, RNA integrity was analysed in 2% agarose gel. RevertAid™ H-Minus First Strand cDNA Synthesis Kit obtained from Thermo Fisher Scientific, India was used for reverse transcription following the manufacturer’s protocols. The synthesized cDNA sample quality was analysed by PCR and stored at − 20 °C until further use.

### Quantitative real-time PCR (RT-qPCR) analysis

The expression of immune related genes comprising of complement component (C3), acute phase protein (transferrin) and a pro-inflammatory cytokine, i.e., IL-1β (interleukin 1-β) were measured and compared with house-keeping gene β-actin (also to check for the integrity of RNA) by Real-time PCR, StepOnePlus Systems (Applied Biosystems, US) with specific pair of primers using (Additional file 1: Table S1) [52–55]. A total reaction volume of 20 µl including 1 µl cDNA (50 ng), 10 µl 2X Maxima SYBR Green/ROX qPCR Master Mix (Thermo Fisher Scientific), 0.5 µl of each specific primer and 8 µl nuclease-free water was maintained for the amplification of the target genes. For each biological replicate of the sample, the master mix was prepared in triplicate, with RT-qPCR for immune related and housekeeping genes performed with a four-step amplification protocol: 10 min at 95 °C (initial denaturation); 40 cycles of 15 s at 95 °C, 30 s at 60 °C, and 30 s at 72 °C (amplification and quantification); 55–95 °C (melting curve) with a 0.10 °C s^−1^ heating rate and a continuous fluorescence measurement and 4 °C cooling. For each primer set, a reaction mixture of negative control was included by omitting the cDNA template. The 2-ΔΔCt method (comparative CT method) following Livak and Schmittgen [56] was used to estimate the target gene expression level and verified by relative standard curve method of Pfaffl [57]. The 2^ΔΔCT values log transformed were subjected to t-test, and the *P* values smaller than 0.05 were considered statistically significant.

### Statistical analysis

The data were transformed arcsin to satisfy the normality and homoscedasticity requirements. These were then subjected to one-way analysis of variances (ANOVA) followed by Duncan’s multiple range test using a statistical package for the social sciences (SPSS) version 24.0. *P*-values smaller than or equal to 0.05 were considered significant.

## Results

### Microbial inoculums improve the water quality of biofloc system

In the first experiment, the effect of microbial inoculum on the water quality of biofloc system was monitored after 35, 70 and 91 days of culture period and was compared with that in the baseline control biofloc group (without microbial inoculums) and negative control group (Table 3). The dissolved NH_4_^+^-N and NO_2_^−^-N concentration (inorganic nitrogen) was lowest in group 2 with [*B. subtilis* (AN2) + *P. fluorescens* (PC3) + *S. cerevisiae* (ATCC-2601)] combinations, followed by group 1 [*B. subtilis* (AN1) + *P. putida* (PB3) + *S. cerevisiae* (ATCC-2601)], group 3 [*B. subtilis* (AN3) + *P. aeruginosa* (PA2) + *S. cerevisiae* (ATCC-2601)], with the baseline control, then negative control containing the highest concentrations (Table 3). However, higher values of NO_3_-N were recorded in biofloc containing groups (microbial inoculums supplemented and baseline control) compared to the negative control group in all sampling days. The TSS and VSS values were observed to be significantly increased in biofloc containing groups as compared to the negative control group. No significant differences were seen in the DO and pH values between the biofloc treatment groups and control groups. In biofloc containing groups, a crude protein content ranging between 28 and 43% was observed during the sampling weeks (Table 3). Maximum values were observed in biofloc treatment groups 2 and 1 followed by group 3 and baseline control. These results indicate that in a biofloc system supplemented with suitable microbial inoculums and maintained at a calculated C/N ratio of 15, water quality is improved.

**Table 3:** Mean values of water quality parameters in treatments, baseline and negative controls (mean ± SE)

Water quality parameters	Sample collection (days)	Negative control	Baseline control	Group 1	Group 2	Group 3
TAN (NH_4_^+^-N + NH_3_) (mg l^−1^)	35	1 ± 0.22^a^	0.62 ± 0.12^b^	0.41 ± 0.14^c^	0.34 ± 0.11^d^	0.59 ± 0.20^b^
	70	0.82 ± 0.11^a^	0.48 ± 0.15^b^	0.17 ± 0.11^d^	0.25 ± 0.14^c^	0.50 ± 0.12^b^
	91	0.95 ± 0.14^a^	0.59 ± 0.11^b^	0.25 ± 0.11^c^	0.21 ± 0.15^c^	0.49 ± 0.12^b^
Nitrite (NO_2_^−^-N) (mg l^−1^)	35	1.37 ± 0.21^a^	0.71 ± 0.16^c^	0.61 ± 0.12^d^	0.57 ± 0.14^d^	0.82 ± 0.24^b^
	70	0.92 ± 0.21^a^	0.57 ± 0.14^b^	0.42 ± 0.11^c^	0.3 ± 0.12^d^	0.52 ± 0.15^b^
	91	0.98 ± 0.14^a^	0.68 ± 0.12^b^	0.18 ± 0.12^c^	0.24 ± 0.14^c^	0.63 ± 0.11^b^
Nitrate (NO_3_^−^-N) (mg l^−1^)	35	2.22 ± 0.12^d^	28.2 ± 2.22^c^	52.5 ± 2.25^b^	64.75 ± 3.32^a^	29.5 ± 1.27^c^
	70	2.35 ± 0.14^c^	43.4 ± 3.16^b^	61.24 ± 2.32^a^	60.2 ± 3.24^a^	36.6 ± 2.21^b^
	91	1.62 ± 0.16^d^	34.3 ± 2.19^c^	59.6 ± 1.25^a^	62.1 ± 2.22^a^	42.2 ± 3.26^b^
Total suspended solids (TSS) (mg l^−1^)	35	50.72 ± 4.31^d^	152.4 ± 3.45^b^	153.5 ± 3.59^b^	160.2 ± 4.21^a^	148.3 ± 4.55^c^
	70	44.32 ± 3.64^c^	155.2 ± 4.28^b^	168.2 ± 4.02^a^	166.3 ± 4.68^a^	152.7 ± 3.54^b^
	91	41.85 ± 3.88^d^	161.82 ± 3.64^c^	171.72 ± 4.27^b^	176.6 ± 3.71^a^	164.22 ± 2.98^c^
Volatile suspended solids (VSS) (mg l^−1^)	35	12.41 ± 3.12^c^	42.7 ± 3.49^b^	48.6 ± 3.15^a^	46.24 ± 3.88^a^	40.3 ± 2.67^b^
	70	14.22 ± 2.87^d^	52.8 ± 4.64^c^	60.7 ± 2.84^a^	56.57 ± 4.28^b^	50.02 ± 3.55^c^
	91	10.82 ± 2.31^c^	50.7 ± 4.72^b^	59.25 ± 4.18^a^	58.67 ± 3.87^a^	52.18 ± 4.55^b^
pH	35	7.4 ± 0.14^a^	7.32 ± 0.22^a^	7.35 ± 0.28^a^	7.25 ± 0.11^a^	7.4 ± 0.14^a^
	70	7.21 ± 0.32^a^	7.4 ± 0.18^a^	7.25 ± 0.15^a^	7.5 ± 0.21^a^	7.1 ± 0.27^a^
	91	7.1 ± 0.18^a^	7.05 ± 0.29^a^	7.25 ± 0.25^a^	7.3 ± 0.18^a^	7.18 ± 0.35^a^
Dissolved Oxygen (mg l^−1^)	35	6.91 ± 0.42^a^	6.7 ± 0.34^a^	6.65 ± 0.38^a^	6.5 ± 0.42^a^	6.7 ± 0.29^a^
	70	7.08 ± 0.36^b^	7.12 ± 0.41^ab^	7.5 ± 0.31^a^	7.47 ± 0.25^a^	7.6 ± 0.38^a^
	91	7.4 ± 0.55^a^	7.12 ± 0.38^ab^	6.95 ± 0.51^b^	7.08 ± 0.47^b^	7.2 ± 0.31^a^
Temperature (°C)	35	28.7 ± 0.12^a^	28.3 ± 0.14^a^	28.3 ± 0.13^a^	28.5 ± 0.12^a^	28.2 ± 0.11^a^
	70	29.2 ± 0.16^a^	28.5 ± 0.11^a^	28.5 ± 0.12^a^	28.4 ± 0.14^a^	28.3 ± 0.14^a^
	91	28.9 ± 0.27^a^	29.4 ± 0.22^a^	30.5 ± 0.18^a^	29.4 ± 0.21^a^	29.2 ± 0.14^a^
Crude protein (% dry weight)	35	0	26.13 ± 3.62^b^	40.2 ± 2.41^a^	42.7 ± 3.22^a^	28.4 ± 4.02^b^
	70	0	31.6 ± 3.56^b^	41.68 ± 2.54^a^	42.97 ± 4.12^a^	30.57 ± 3.28^b^
	91	0	29.5 ± 2.98^b^	42.5 ± 4.28^a^	43.1 ± 2.48^a^	30.8 ± 2.85^b^

Significant differences between control and treatment groups at each sampling point are indicated with different superscript, a denotes highest value followed by b, c and d

### Microbial inoculums regulate bacterial composition in both in vivo and in vitro conditions

There have been numerous investigations that *Bacillus* and *Pseudomonas* species may contribute to floc formation and nutrition, improve water quality and inhibits pathogen adherence and colonization in the gastrointestinal tract, which results in enhanced growth performance and immune response of cultured animals [3, 5, 58, 59]. Adherence and colonization efficiency of *Bacillus* and *Pseudomonas* were investigated by estimating their respective count in biofloc and gut samples. Treatment group 2 appeared to have significant effect on bacterial count followed by group 1, group 3, baseline and negative controls. In biofloc samples, higher levels of total plate count (TPC) and *Bacillus* and *Pseudomonas* count were observed in 35, 70 and 91 days (Tables 4, 5). In parallel with biofloc samples, the gut samples of *H. fossilis* had higher TPC, which also included higher *Bacillus* and *Pseudomonas* counts. Similarly, the highest values were recorded in group 2 followed by group 1, group 3, baseline and negative controls (Tables 6, 7). The bacterial counts in the biofloc and gut samples of group 3 were not significantly different from the baseline control group. Overall, the results suggest that the microbial inoculum composition has determining role in adherence and colonization of bacterial strains in both biofloc and gut microbiota of *H. fossilis*.

**Table 4:** Mean values of total plate count in biofloc samples of treatments, baseline and negative control groups (mean ± SE)

Sample collection (days)			Treatment groups		
	Negative control	Baseline control	Group 1	Group 2	Group 3
7	4.4 × 10^–3^	6.4 × 10^–3^	9.5 × 10^–3^	7.1 × 10^–3^	5.8 × 10^–3^
14	5.2 × 10^–3^	6.1 × 10^–3^	1.2 × 10^–4^	6.7 × 10^–3^	5.5 × 10^–3^
21	2.8 × 10^–3^	2.1 × 10^–4^	4.6 × 10^–5^	3.2 × 10^–5^	7.2 × 10^–4^
28	7.2 × 10^–3^	1.7 × 10^–5^	2.7 × 10^–7^	2.6 × 10^–6^	1.1 × 10^–6^
35	1.7 × 10^–4^	2.7 × 10^–6^	7.1 × 10^–7^	2.1 × 10^–7^	8.8 × 10^–6^
42	1.3 × 10^–3^	1.8 × 10^–6^	6.5 × 10^–7^	1.5 × 10^–7^	1.8 × 10^–6^
49	7.8 × 10^–3^	1.3 × 10^–6^	2.1 × 10^–7^	1.9 × 10^–7^	4.6 × 10^–6^
56	2.2 × 10^–4^	1.1 × 10^–7^	3.2 × 10^–8^	1.3 × 10^–8^	1.5 × 10^–7^
63	1.1 × 10^–3^	2.5 × 10^–7^	2.1 × 10^–8^	1.9 × 10^–8^	1.6 × 10^–8^
70	2.1 × 10^–3^	1.6 × 10^–7^	2.9 × 10^–8^	2.2 × 10^–8^	1.9 × 10^–8^
77	1.9 × 10^–4^	3.2 × 10^–7^	1.8 × 10^–8^	1.7 × 10^–8^	1.1 × 10^–7^
84	1.4 × 10^–3^	1.9 × 10^–7^	3.5 × 10^–8^	2.6 × 10^–8^	2.9 × 10^–7^
91	1.6 × 10^–3^	1.3 × 10^–7^	1.3 × 10^–8^	1.1 × 10^–8^	2.1 × 10^–7^

**Table 5:** Mean values of *Bacillus* and *Pseudomonas* count in biofloc samples of treatment, baseline and negative control groups (mean ± SE)

Sample collection (days)					Treatment groups					
	Negative control		Baseline control		Group 1		Group 2		Group 3	
	*Bacillus* count	*Pseudomonas* count	*Bacillus* count	*Pseudomonas* count	*Bacillus* count	*Pseudomonas* count	*Bacillus* count	*Pseudomonas* count	*Bacillus* count	*Pseudomonas* count
35	–	–	5.4 × 10^–3^	4.8 × 10^–3^	5 × 10^–6^	2.1 × 10^–6^	7.1 × 10^–7^	3.8 × 10^–7^	5.1 × 10^–5^	1.4 × 10^–5^
70	–	–	1.8 × 10^–4^	5.3 × 10^–3^	4.1 × 10^–7^	1.1 × 10^–6^	6.5 × 10^–8^	4.2 × 10^–8^	2.5 × 10^–6^	2.2 × 10^–5^
91	–	–	1.2 × 10^–4^	1.5 × 10^–4^	1.8 × 10^–7^	2.4 × 10^–7^	3.2 × 10^–8^	2.5 × 10^–8^	3.9 × 10^–6^	1.8 × 10^–5^

**Table 6:** Mean values of total plate count (TPC) from gut samples of *Heteropneustes fossilis* in treatment, baseline and negative control groups (mean ± SE)

Sample collection (days)			Treatment groups		
	Negative control	Baseline control	Group 1	Group 2	Group 3
35	2.1 × 10^–6^	1.4 × 10^–6^	2.4 × 10^–8^	2.6 × 10^–8^	3.1 × 10^–6^
70	2.26 × 10^–6^	2.4 × 10^–7^	2.8 × 10^–8^	2.9 × 10^–8^	1.8 × 10^–7^
91	1.2 × 10^–6^	1.3 × 10^–7^	1.65 × 10^–8^	1.8 × 10^–8^	2.4 × 10^–7^

**Table 7:** Mean values of *Bacillus* and *Pseudomonas* count from gut samples of *Heteropneustes fossilis* in treatment, baseline and negative control groups (mean ± SE)

Sample collection (days)					Treatment groups					
	Negative control		Baseline control		Group 1		Group 2		Group 3	
	*Bacillus* count	*Pseudomonas* count	*Bacillus* count	*Pseudomonas* count	*Bacillus* count	*Pseudomonas* count	*Bacillus* count	*Pseudomonas* count	*Bacillus* count	*Pseudomonas* count
35	–	–	2.7 × 10^–4^	1.2 × 10^–4^	1.4 × 10^–7^	1.9 × 10^–6^	1.5 × 10^–8^	1.4 × 10^–7^	2.1 × 10^–6^	1.02 × 10^–6^
70	–	–	2.2 × 10^–4^	3.1 × 10^–5^	2.4 × 10^–7^	2.1 × 10^–7^	2.5 × 10^–8^	2.2 × 10^–8^	2.7 × 10^–6^	1.5 × 10^–6^
91	–	–	3.4 × 10^–4^	2.5 × 10^–5^	1.2 × 10^–8^	3.3 × 10^–7^	2.8 × 10^–8^	1.5 × 10^–8^	1.9 × 10^–7^	1.4 × 10^–7^

### Effect of microbial inoculums on gut histomorphology and growth performance of *H. fossilis*

**Fig. 1:**
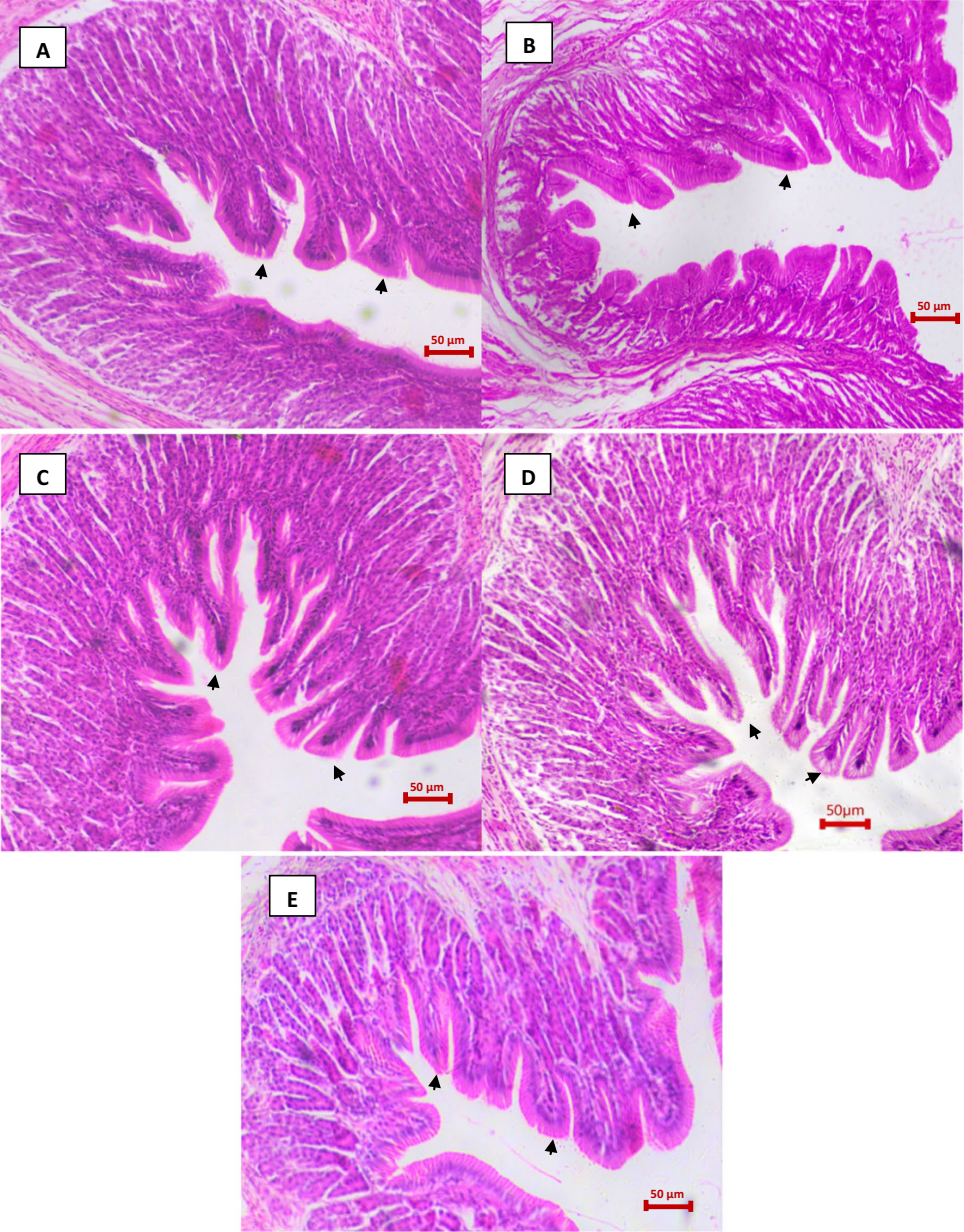
Microphotographs of histological section (H&E) from gut samples of *Heteropneustes fossilis*. **A** Negative control; **B** Baseline control; **C** Group 1; **D** Group 2; and **E** Group 3. The arrowhead in figures (**A**–**E**) represents the modulation in gut morphology and villi length of fish collected from treatment, baseline and negative control groups

The efficacies of microbial inoculums in enhancing gut histomorphology and growth of *H. fossilis* were investigated. The histological analysis demonstrates that the gut morphology of biofloc treatment groups were better when compared with the control groups (Fig. 1a–e). The treatment fish have higher villi length and wider intestinal walls compared to the control fish. In addition, the microvilli have increased height, width, and relative absorptive area in the treatment group of fish (Fig. 1c–e).

The growth performance of *H. fossilis* reared in biofloc containing systems supplemented with microbial inoculums was significantly higher when compared to fish cultivated in the baseline control (biofloc system without microbial inoculums) and negative control (clear water system) (Table 8). The weight gain, final body weight, weekly weight gain and specific growth rate of fish reared in microbial inoculums supplemented treatment groups were significantly higher compared to the baseline and negative control groups. Additionally, the biofloc maintained with microbial inoculums, especially in groups 2 and 1 resulted in a significantly lower FCR (food conversion ratio) compared to group 3, baseline and negative controls (Table 8).

**Table 8:** Mean values of growth parameters in treatment, baseline and negative control groups (mean ± SE)

Parameters			Treatment groups		
	Negative control	Baseline control	Group 1	Group 2	Group 3
Initial weight (g)	1.72 ± 0.27^a^	1.72 ± 0.27^a^	1.72 ± 0.27^a^	1.72 ± 0.27^a^	1.72 ± 0.27^a^
Final weight (g)	5.2 ± 2.52^d^	6.45 ± 2.27^cd^	7.9 ± 2.42^b^	8.7 ± 2.18^a^	6.85 ± 2.34^c^
Weight gain (g)	3.45 ± 1.54^e^	4.71 ± 1.85^d^	6.18 ± 1.24^b^	6.95 ± 1.08^a^	5.13 ± 2.14^c^
Weekly weight gain (g wk^−1^)	0.27 ± 0.06^c^	0.36 ± 0.04^b^	0.48 ± 0.04^a^	0.53 ± 0.08^a^	0.39 ± 0.05^b^
Specific growth rate (% day^−1^)	3.79 ± 1.62^e^	5.18 ± 2.41^cd^	6.79 ± 1.27^b^	7.64 ± 1.09^a^	5.64 ± 2.24^c^
Feed conversion ratio (FCR)	1.56 ± 0.16^a^	1.47 ± 0.22^b^	1.27 ± 0.24^d^	1.21 ± 0.18^d^	1.38 ± 0.27^c^

Significant differences between control and treatment groups at each sampling point are indicated with different superscript, a denotes highest value followed by b, c and d

**Fig. 2:**
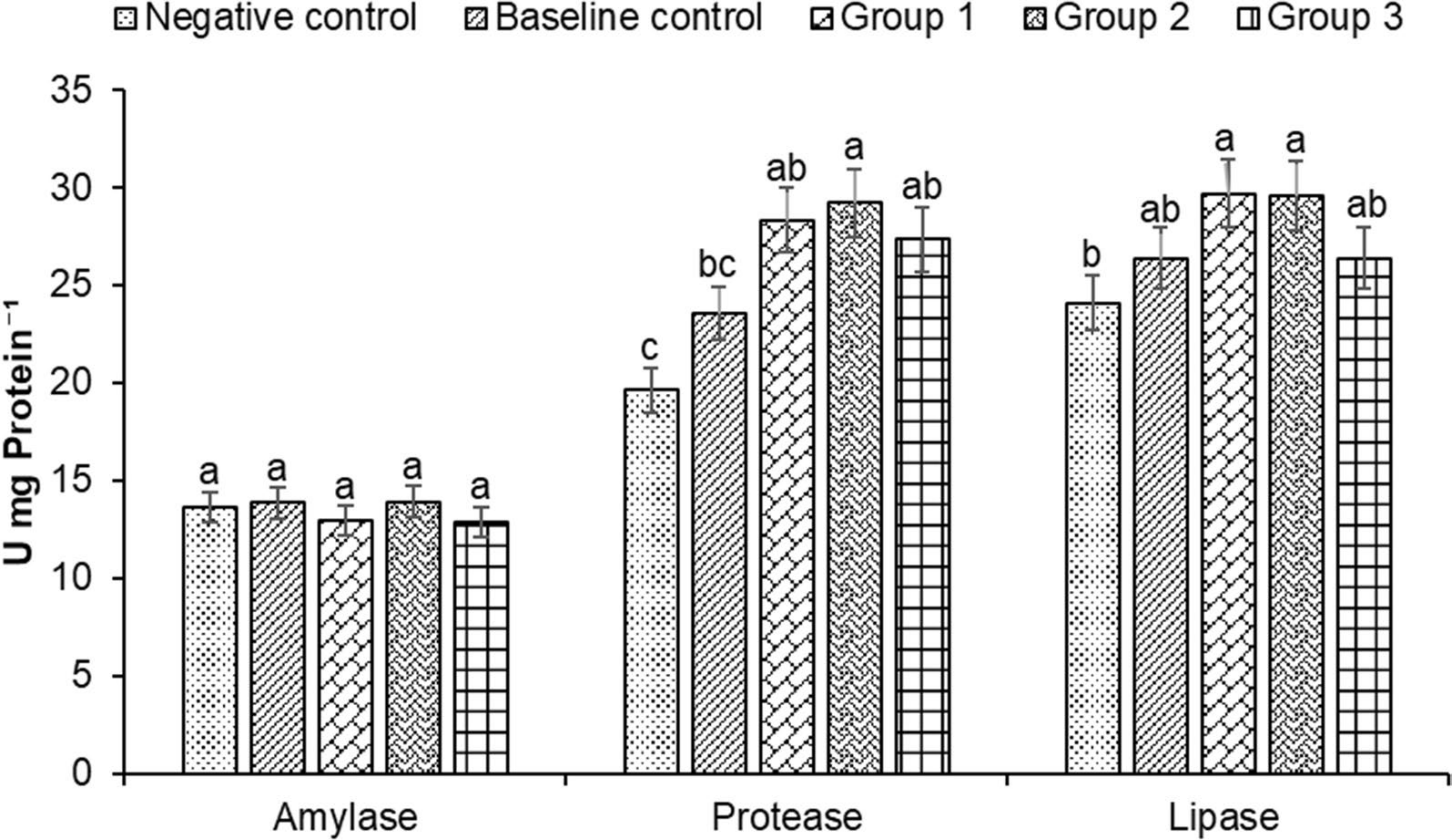
Changes in amylase, protease and lipase activity in the gut of *Heteropneustes fossilis* in biofloc based culture system. The results are the mean ± SE (n = 3) and the vertical bars with different superscripts (a denotes highest value followed by b, c and d) indicate significant differences between treatment groups (*P* < 0.05)

Among the control and treatment groups, there were no significant differences in the amylase value. The amylase value ranged from 12.86 ± 0.76 to 13.84 ± 0.11 U mg Protein^−1^ between the control and treatment groups (Fig. 2). Protease activity was shown to be significantly different across the treatment and control groups, with highest value observed in group 2 followed by groups 1, 3, baseline, then negative control (Fig. 2). Similarly, the lipase activity was found to be significantly higher in groups 1 and 2, with lower values recorded in group 3, baseline and negative control (Fig. 2). These results suggest that the addition of a suitable combination of microbial inoculums would be an efficient method to enhance the biofloc-driven culture environment and nutrition for the fish, resulting in enhanced growth performance of *H. fossilis* [26, 60–62].

### Microbial inoculums enhance antioxidant defense of *H. fossilis*

**Fig. 3:**
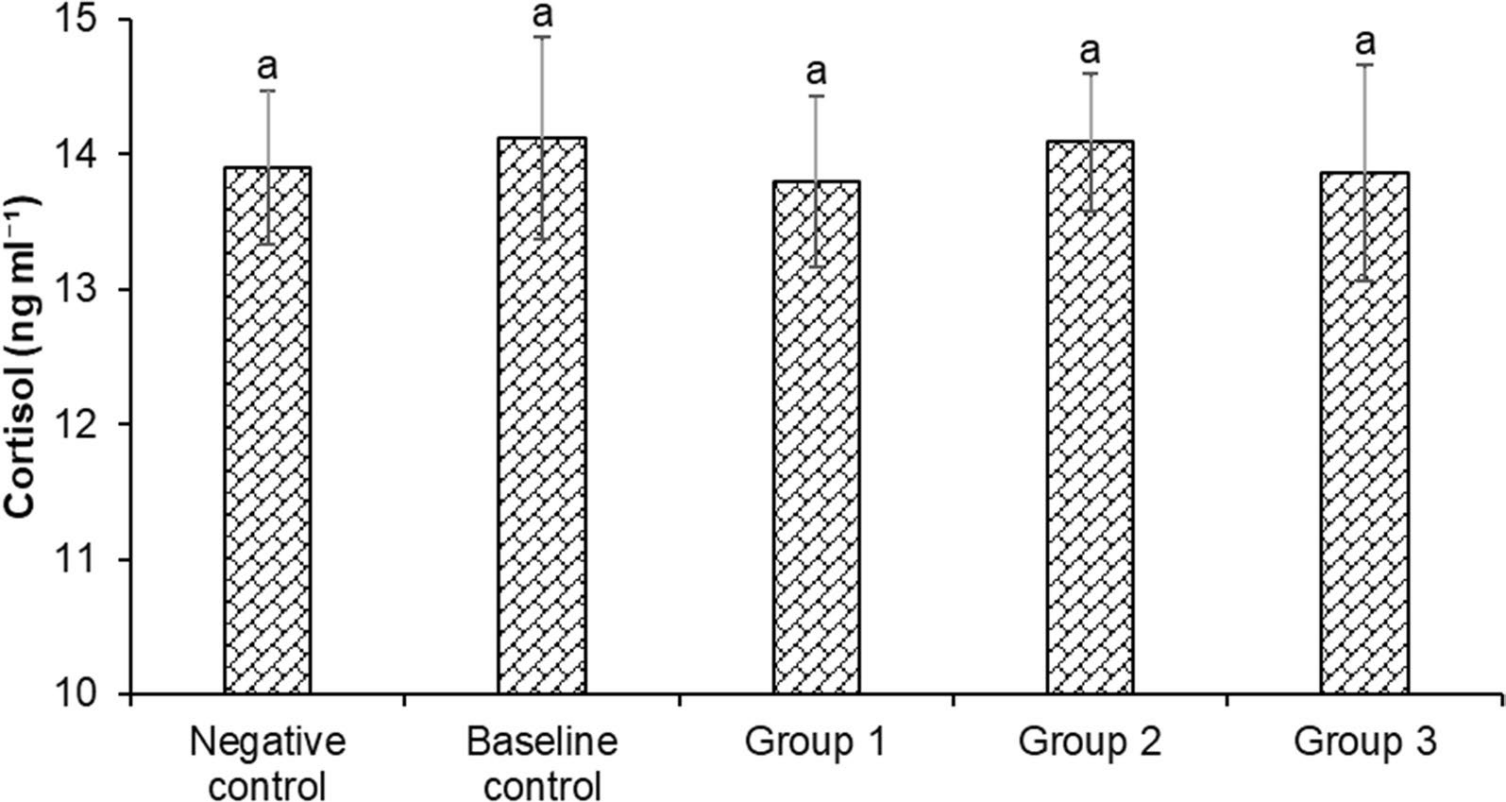
Changes in serum cortisol of *Heteropneustes fossilis* in biofloc based culture system. The results are the mean ± SE (n = 3) and the vertical bars with different superscripts indicate significant differences between treatment groups (*P* < 0.05)

In the next experiment, we investigate the mechanism of action from microbial inoculums in enhancing the growth-promoting effect on *H. fossilis*. The findings revealed that cortisol concentration did not differ significantly among the treatment and control groups and was found to be at basal or resting levels in the cultivated fingerlings. The cortisol level ranged from 13.86 ± 0.80 to 14.12 ± 0.75 ng ml^−1^ between the control and treatment groups (Fig. 3).

**Fig. 4:**
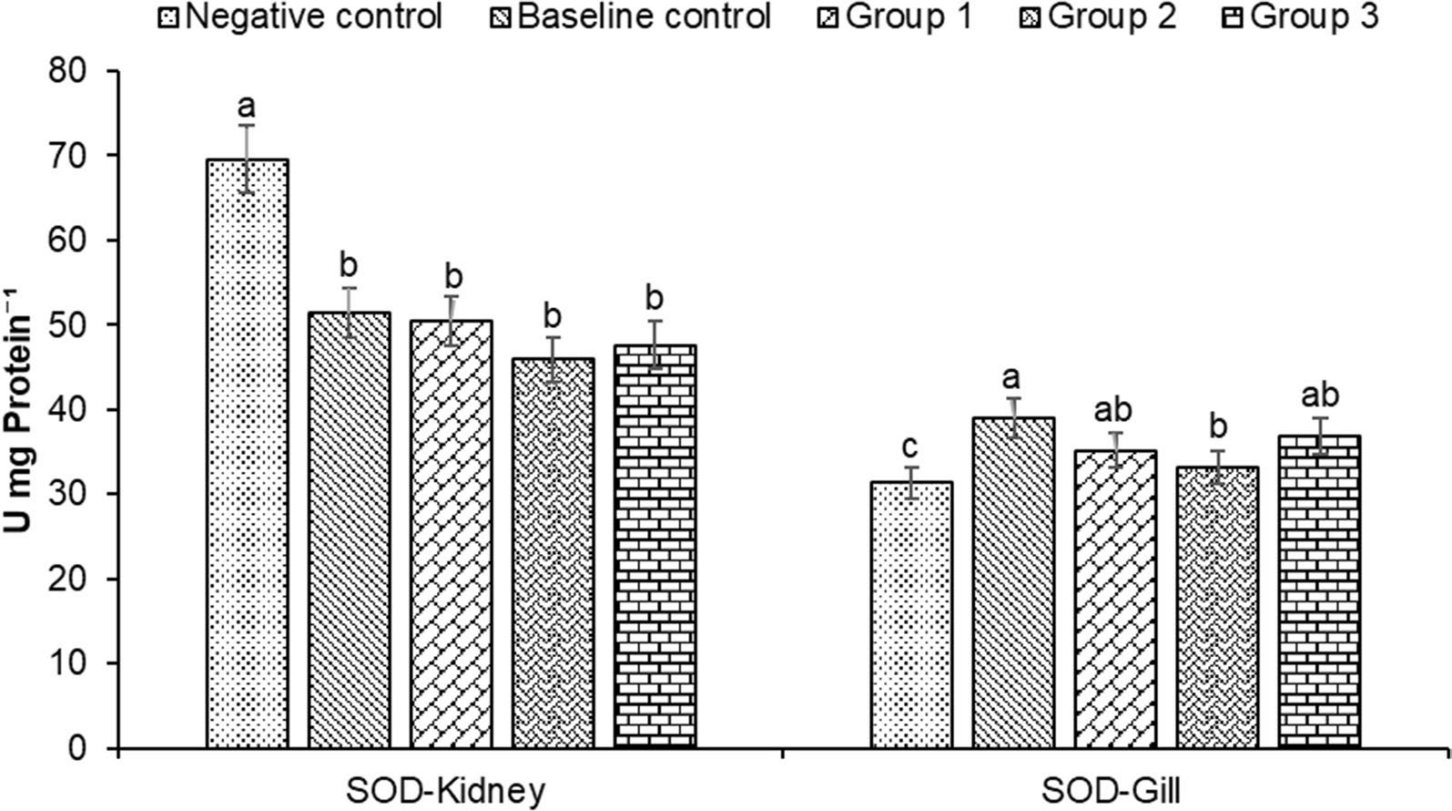
Changes in superoxide dismutase (SOD) in kidney and gill of *Heteropneustes fossilis* in biofloc based culture system. The results are the mean ± SE (n = 3) and the vertical bars with different superscripts (a denotes highest value followed by b, c and d) indicate significant differences between treatment groups (*P* < 0.05)

**Fig. 5:**
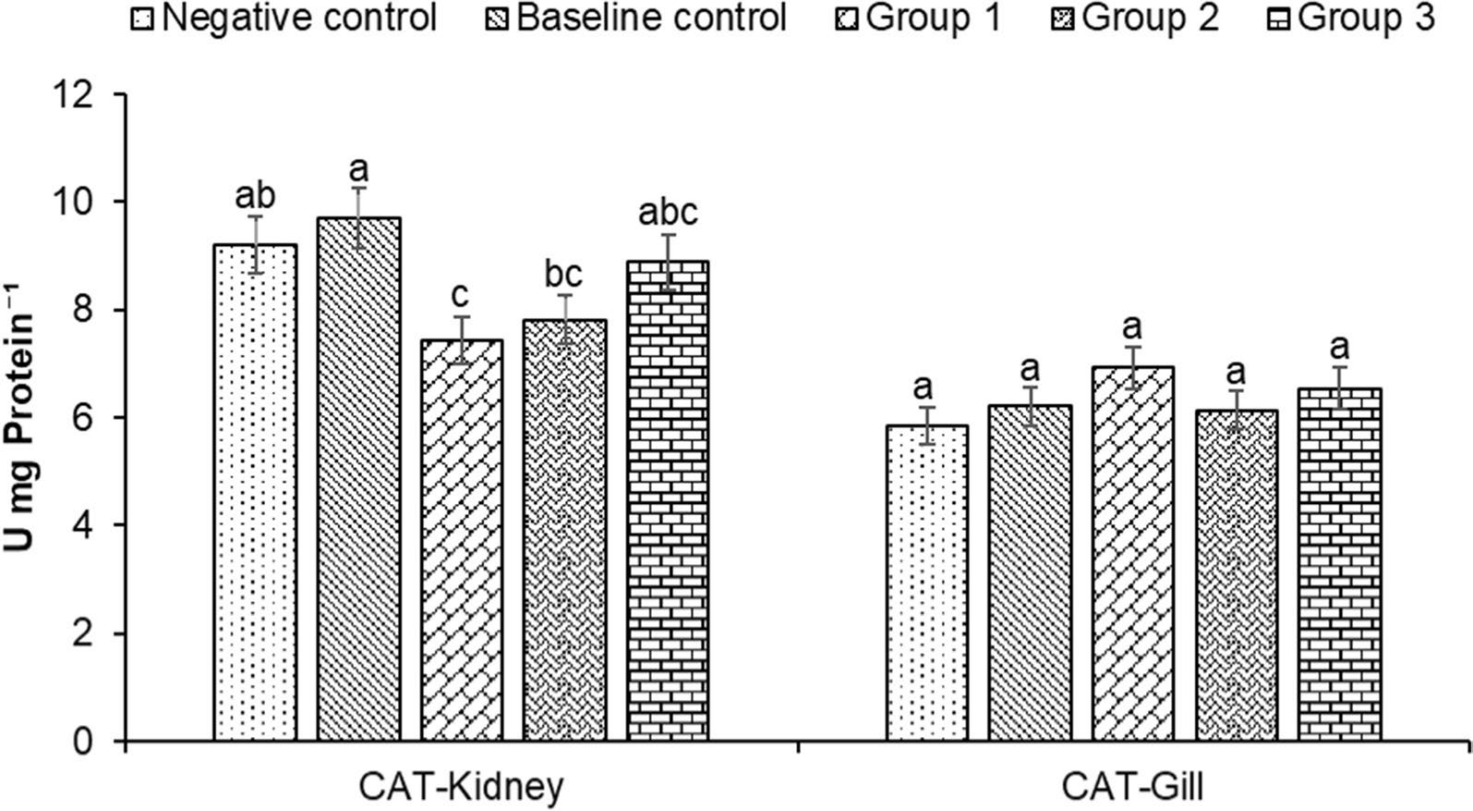
Changes in catalase (CAT) in the kidney and gill of *Heteropneustes fossilis* in biofloc based culture system. The results are the mean ± SE (n = 3) and the vertical bars with different superscripts (a denotes highest value followed by b, c and d) indicate significant differences between treatment groups (*P* < 0.05)

Superoxide dismutases (SOD) and catalase (CAT) located within cellular cytosolic and mitochondrial compartments are primary antioxidant defense components in fish responsible for the toxic superoxide anion radical’s detoxification. In our study, the SOD activity in fish kidneys was significantly higher in the negative control group compared to other groups. In contrast, significantly higher SOD values were recorded in baseline control fish gills followed by groups 3, 1 and 2, with the lowest values found in negative control group (Fig. 4). The CAT activity was significantly elevated in the kidney of control group fishes, with high levels of CAT value recorded in baseline (9.69 ± 0.56 U mg Protein^−1^) and negative control (9.19 ± 0.53 U mg Protein^−1^) fish kidneys (Fig. 5). However, in fish gills higher CAT activity was found in treatment groups, followed by the baseline and negative controls (Fig. 5). Highest values were found in group 1 (6.92 ± 0.40 U mg Protein^−1^) followed by group 3 (6.54 ± 0.38 U mg Protein^−1^), baseline control (6.21 ± 0.35 U mg Protein^−1^), group 2 (6.14 ± 0.35 U mg Protein^−1^) and negative control (5.84 ± 0.34 U mg Protein^−1^). These results indicate that microbial inoculums mediated decreased antioxidant response, which appeared to be at least in part due to decreased oxidative stress in treatment groups. However, this observation requires further validation.

### Modulation of health and immunity of *H. fossilis* by microbial inoculums in biofloc system

**Fig. 6:**
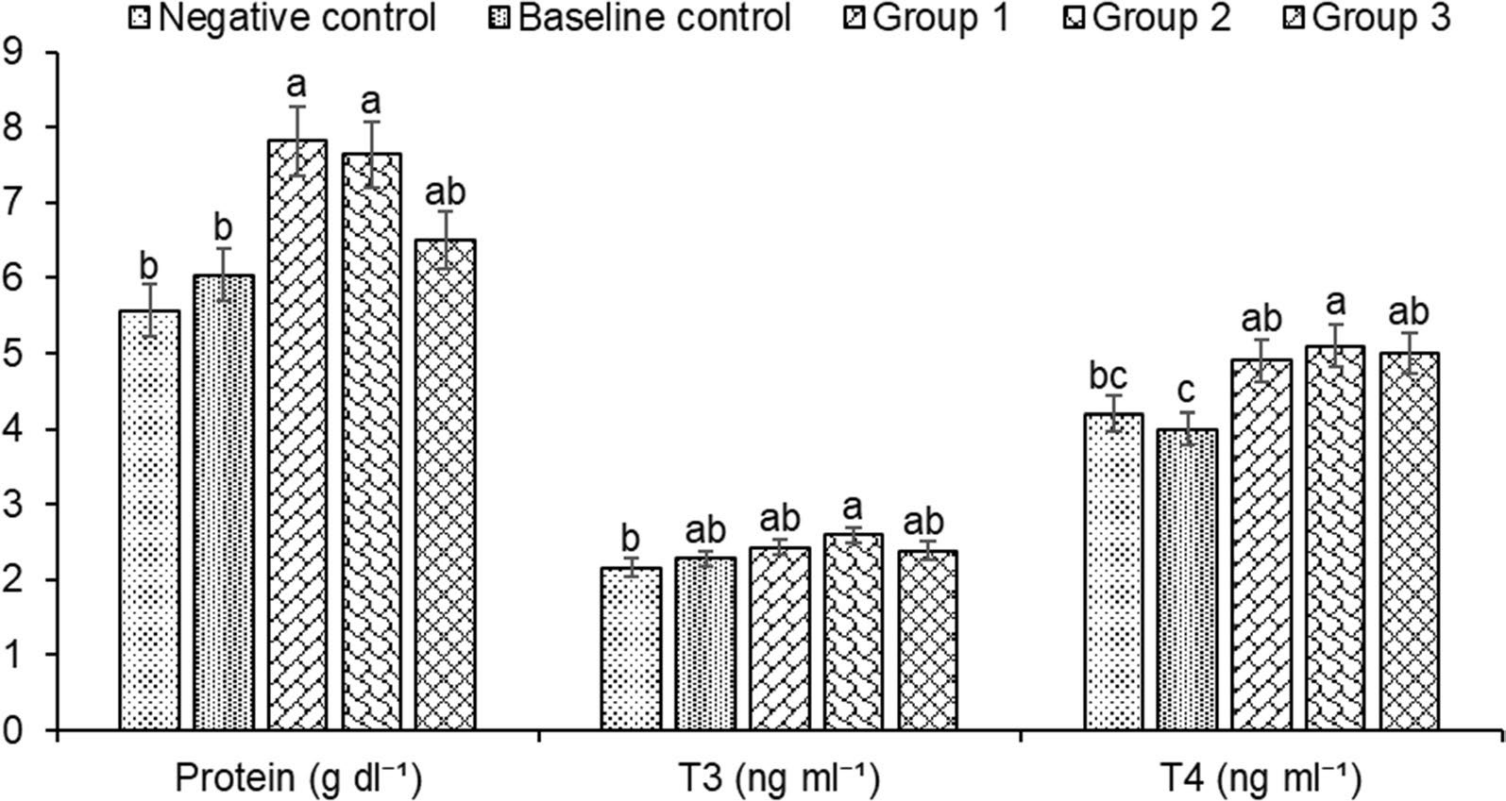
Changes in protein, T3 (Triiodothyronine) and T4 (Thyroxine) in the serum of *Heteropneustes fossilis* in biofloc based culture system. The results are the mean ± SE (n = 3) and different letters (a denotes highest value followed by b, c and d) indicate significant differences between treatment groups (*P* < 0.05)

**Fig. 7:**
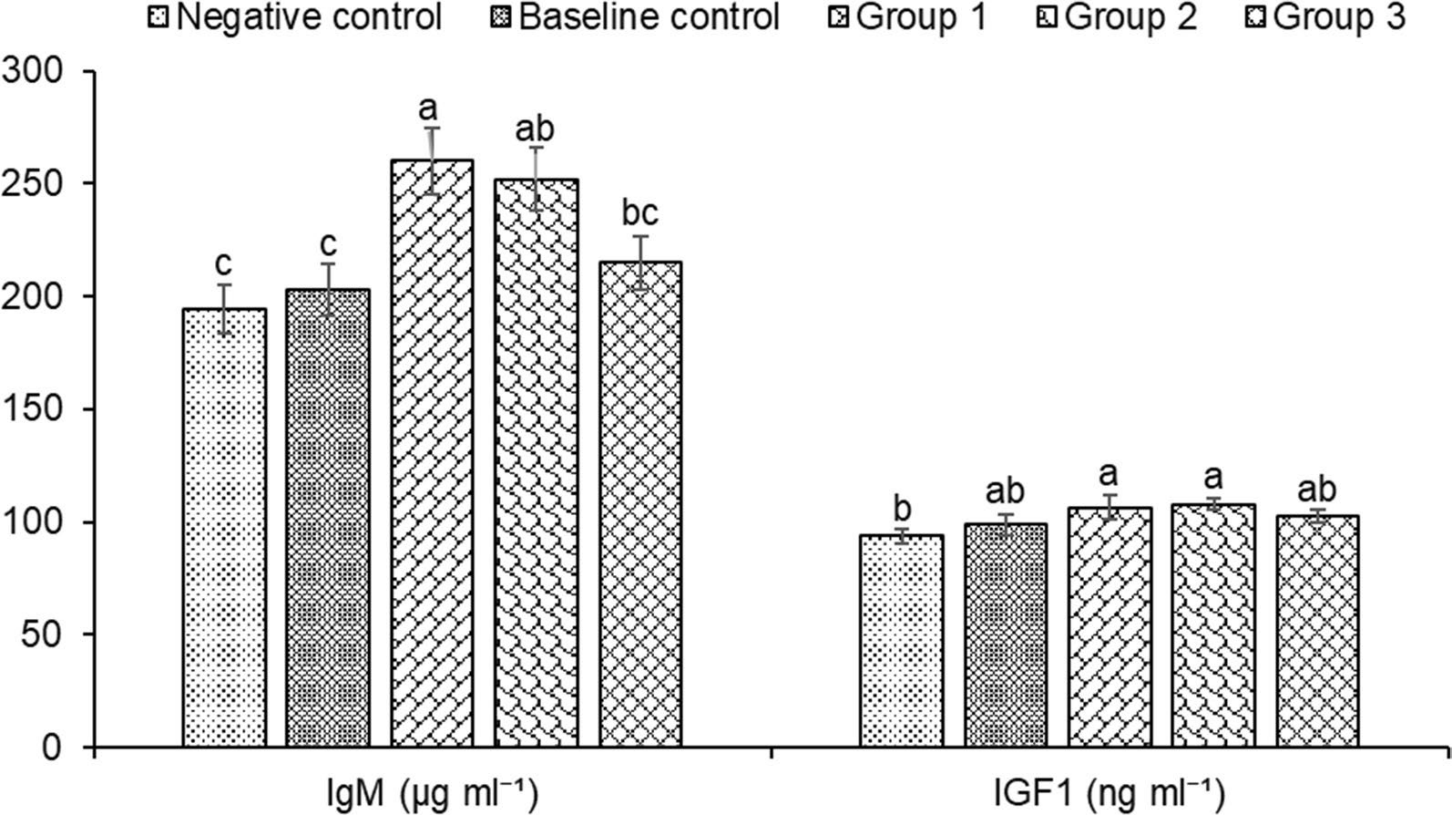
Changes in IgM and IGF1 in the serum of *Heteropneustes fossilis* in biofloc based culture system. The results are the mean ± SE (n = 3) and the vertical bars with different superscripts (a denotes highest value followed by b, c and d) indicate significant differences between treatment groups (*P* < 0.05)

Enzymatic and molecular assays were used to assess the role of microbial inoculums on the health and immunity of *H. fossilis*. We found that biofloc groups supplemented with microbial inoculums have significantly increased levels of T3 (Triiodothyronine) and T4 (Thyroxine) in fish serum (Fig. 6). The highest levels of T3 and T4 activity were observed in the treatment groups, followed by the baseline and negative controls. The total protein concentrations observed were increased and significantly different across the various treatment groups compared to the controls, with the maximum values observed within groups 1–3 (Fig. 6) with treatment group 1 showing the highest total protein content. The activity of IgM in the serum of biofloc treatment fingerlings (1–3 groups) was found to be significantly increased when compared to the fingerlings of control group (Fig. 7). Results showed that in the biofloc treatments, group 1 fingerlings exhibited significantly increased IgM activity, with lower values recorded in group 2, 3 baseline and negative controls. The insulin-like growth factor-1 (IGF1), which is positively correlated with the growth rate of fish, was significantly increased in the microbial inoculum treatment groups (groups 1–3) compared to the control groups (Fig. 7).

**Fig. 8:**
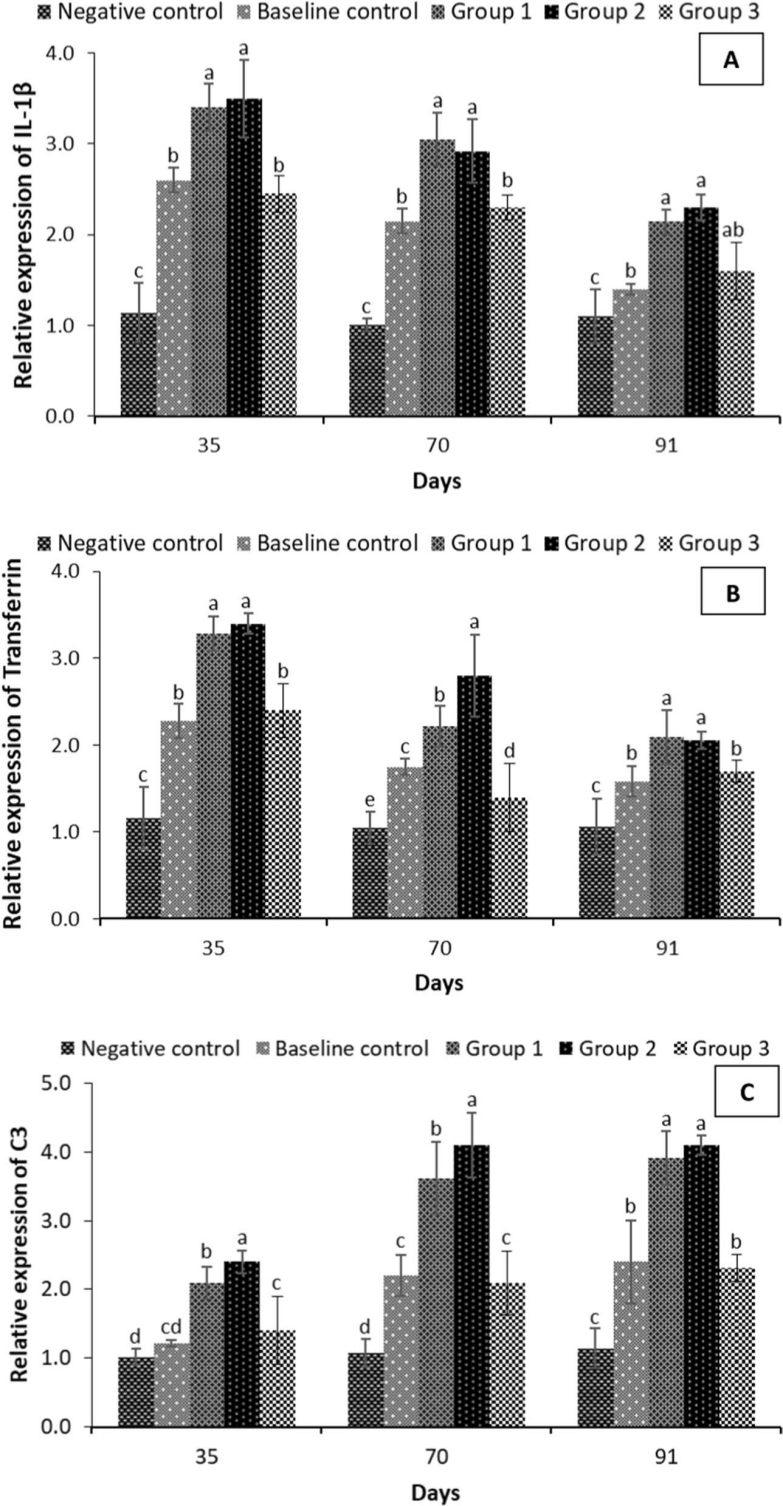
Fold change in immune gene expression of *Heteropneustes fossilis* fingerlings in different treatment groups. **A** Expression of Interleukin-1β (IL-1β); **B** Transferrin and **C** C3 complement system as determined by quantitative real-time PCR. The expression level in the control group (− microbial inoculums and carbon addition) was regarded as 1.0 and thereby the expression ratio of the baseline control group (− microbial inoculums) and treatments (+ microbial inoculums and carbon addition) was expressed in relation to the control group. The results are the mean ± SE (n = 3) and the vertical bars with different superscripts (a denotes highest value followed by b, c and d) indicate significant differences between treatment groups (*P* < 0.05)

To analyse the transcriptional changes influenced by the microbial inoculum supplementation, the in vivo temporal expression of complement component (C3), acute phase protein (transferrin) and a pro-inflammatory cytokine, i.e., IL-1β (interleukin 1-β) were measured and compared to the control groups. The qPCR results highlighted that complement component C3, transferrin and interleukin 1β (IL-1β) genes exhibited differential expression profiles in the microbial inoculum treatment groups compared to the control group. The expression of the acute phase protein (transferrin) and a pro-inflammatory cytokine, IL-1β were significantly unregulated 35- and 70-days post-treatment in group 3 followed by 1, 3 and baseline control groups (Fig. 8a, b). Moreover, the transcription of C3, complement system central component, was significantly unregulated at 70 and 90 days post-treatment, with the highest values recorded (~ 4 folds or more) in groups 2 and 1 (Fig. 8c). The results suggest that microbial inoculum with specific strain combinations may provide potential benefits towards the host, leading to improvements in health and immunity of *H. fossilis* fingerlings.

## Discussion

Biofloc technology is an innovative aquaculture system with great potential towards sustainable fish production. The approach is based on a zero to minimal water exchange system with a principle of waste nutrient recycling into microbial biomass that can be harvested and processed into feed ingredients or used in situ by farmed animals [63]. Biofloc-driven aquaculture production has the potential to realize various United Nations Sustainable Development Goals (SDGs) such as goal 1—no poverty; goal 2—zero hunger; goal 5—gender equality; goal 12—responsible consumption and production; and goal 14—life below water [20, 64]. The biofloc-driven approach enables efficient use of nutrients within the system and optimizes water use efficiency which can subsequently reduce the cost of production for enhanced livelihood among smallholder aquaculture farmers [60, 65]. However, the biofloc technology has only been standardized for 3–4 aquatic species—tilapia, shrimp, and *Pangasius* sp.—with an urgent need to investigate the feasibility of the technology for other indigenous high-valued fish species. Additionally, standardization of microbial inoculums that form the starter culture is needed to develop effective and reliable biofloc for efficient conversion of waste and to improve farming productivity. In this study, different combinations of microbial inoculum with probiotics and bioremediation properties were investigated. We found that microbial inoculums with select compositions resulted in optimal biofloc development, and provided optimal waste treatment characteristics, such as the removal of ammonia and other nitrogenous compounds. Subsequently, the microbial inoculums were observed to establish a beneficial microbiome within the flocs and host alike, leading to improvements to gut histomorphology, growth performance, anti-stress and antioxidant properties, and protective immunity in *H. fossilis.*

Our main motivation for using a microbial consortium was to exploit advantages of the different species and improve floc quality and health of the cultured animal [23]. The microbial consortium used in the study displayed different properties; for instance, *Bacillus subtilis* strains (AN1, AN2 and AN3) exhibits immunostimulatory properties. The *Pseudomonas* species (*putida* PB3, *fluorescence* PC3 and *aeruginosa* PA2) have floc formation and bioremediation properties. Lastly, the yeast species *Saccharomyces cerevisiae* (ATCC 2601) modulate EPS (extracellular polymeric substances) and floc formation and exhibits bioremediation properties [66–68]. However, as most synthetic microbial consortia are competitive, a primary challenge in their design is to avoid the dominance of one species over another, due to a shorter doubling time or production of substances that are inhibitory to the other species. Conventionally, titration of the inoculum ratio and optimization of growth conditions (such as pH and temperature) can be exploited to maintain coexistence. In this study, we avoided these complications by building a mutualistic co-culture. At first, we did growth compatibility assay, to check whether the microbial species exert antimicrobial activity against other used strains. We also explored other strategies to avoid microbial competition. Experiments were designed to investigate whether the assigned properties of individual microbial species in microbial consortium is hampered or not [1, 18]. The study highlighted microbial species that don’t inhibit the growth and beneficial roles of other species and acts synergistically to improve the biofloc and health of cultured host animals. Hence, the microbial consortium concept might be generally applicable in best quality flocs production for use in sustainable aquaculture.

The main principle of biofloc technology is to cultivate aquatic animals in high density settings whilst maintaining optimum water quality with minimal or no exchange of water. This is achieved by nutrient recycling, particularly for nitrogenous compounds, into microbial biomass which could be harvested and processed into feed ingredients or utilized by cultivated animals in situ [24, 27, 29]. In this study, the treatment groups containing microbial inoculum had significantly improved water quality parameters and were found to produce higher growth performance and immune response in fish [69–71]. Improvements in water quality were also seen in the reduction of NH_4_^+^-N + NH_3_ (TAN) and NO_2_^−^-N (nitrite) concentrations in the treatment groups after 35, 70 and 91 days compared to the negative control. The biofloc system is supported by the aggregation of heterotrophic microorganisms, which are associated with bioremediation processes [40, 61, 69, 72–76]. Therefore, the biofloc influenced by the addition of select microbial inoculums may create beneficial microbiomes resulting in improved water quality parameters within the biofloc-associated cultivation system.

The colonization of introduced bacteria new to the system into both flocs and intestines of fish could serve a potential indicator of biofloc system functioning, as these species and their ecological associations regulate the structural composition of the flocs, along with water quality and the health of cultured animals [23, 77]. The microbiota present also has an essential role in regulating feeding behaviour, and can influence the appetite and food intake of the host [78, 79]. The composition of the aquatic and gut microbiomes has be shown to be shaped by exogenous factors including rearing environment, dietary microbial supplementation, and abiotic factors [80–82]. The total plate count (TPC) as well as *Bacillus* and *Pseudomonas* counts in biofloc and gut samples were significantly modulated in response to the introduction of microbial inoculum supplementation*.* The addition of inoculums consisting of different microbial strains were shown to significantly enhance the presence of beneficial *Bacillus* and *Pseudomonas* groups, with the group 2 treatment inoculum containing *B. subtilis* (AN2) + *P. fluorescens* (PC3) + *S. cerevisiae* (ATCC-2601) providing the greatest effect on the bacterial count compared to other groups. This indicates that the effect of microbial inoculums on bacterial abundance is strain-specific and has a profound effect on the development of the beneficial aspects of the biofloc system.

Improved feed utilization and growth performance in cultivated fish after microbial supplementation can be attributed to probiotic effects promoting beneficial intestinal microflora. The enhanced nutrition and absorption efficiency of indigestible components through hydrolytic enzymes, including amylases, lipase and proteases is likely improved through the introduction of the biofloc-based probiotic activity [83, 84]. Reports also suggest that enhanced nutrition and absorption efficiency is generally achieved through intestinal modulation, in general through changes in villous morphology [85]. In the present study, the villi length was increased in the gut of fish supplemented with microbial inoculums. In addition, the microbial inoculums have been shown to improve the growth performance and digestive enzyme activity including amylase, protease and lipase activities in the gut samples of *H. fossilis*. A possible mechanism is that the probiotics modulate the gut bacterial diversity and morphology (villi length), resulting in increased surface area for absorption leading to improved dietary nutrient utilization, and ultimately higher growth performance in biofloc treated cultivated fish. Similar observations have been reported in tilapia (*Oreochromis niloticus)* and trout (*Oncorhynchus mykiss*), where probiotic supplementation has been shown to improve intestinal morphology, digestive enzyme activities, nutrient absorption and growth performance [86, 87].

Stress-induced immune function impairment has been widely studied in wild and cultured fish and is often associated with decreased health and survival [88], with water quality parameters a major driver of fish response to recovery dynamics and stress [89]. Cortisol, a principal corticosteroid, is released in response to stress and disease, and plays an essential role in mediating adaptive physiological, metabolic and behavioral adjustments [90, 91]. Cortisol levels in fish can be used as an indicator for health status, as prolonged increases in cortisol levels have been associated negatively with growth, immunity, disease resistance, development and reproduction [91]. In addition, oxidative stress is closely linked with immune response in fish, with excessive production of RNS (reactive nitrogen species) and ROS (reactive oxygen species) during stress leading to a highly cytotoxic metabolic environment, which can result in organ damage and imbalances in the immune response [92–94]. In our study, treatment and control groups did not appear to affect cortisol levels. A possible explanation is the biofloc present in the baseline control and treatment groups created an environment with suitable water quality, mitigating waterborne stressors and leading to low levels of cortisol observed in the *H. fossilis* fingerlings. Additionally, the negative control group has approximately 60% of the water volume exchanged daily, which might have reduced the build-up of stressors in the system.

A stochastic pattern of interaction was observed in treatment and control groups on the antioxidant defense system. The findings suggest that microbial supplementation exhibited enhanced SOD and CAT activity (antioxidant enzyme) in *H. fossilis*. The enhanced antioxidant activity might be responsible for the detoxification and clearance of free radicals from the biofloc treatment groups, and impart protection from oxidative stress [95]. These results were consistent with the known beneficial roles of microbial species, when supplemented in the cultivation system, helps induce anti-stress and antioxidant responses in fish [96, 97].

Microbial-based compounds and their derivatives have been linked with enhanced immune response in fish [51, 98, 99]. There exists a correlation between reduced stress and enhanced antioxidative response with immune response in animals [8, 98, 100]. Microbial compounds have also been known to induce thyroid hormone activity, including thyroxine (T4) and triiodothyronine (T3) and IGF1 (insulin-like growth factor-1), which have been positively correlated with the growth rate in fish [101, 102]. Thus, it has been proposed that monitoring T3, T4 and IGF1 levels might serve as a potential indicator of growth, development, metabolism, and reproduction in fish. In the treatment groups, the T3, T4, IGF1 and IgM levels were enhanced in *H. fossilis* fingerlings when compared to the control groups, suggesting that the microbial inoculum in the biofloc group may improve both growth activity (T3, T4 and IGF1) and immune response (IgM) of fish. This may be due to the microbial inoculums promoting the colonization of beneficial microbes in flocs and gut, leading to the observed improvements in the digestive and antioxidative enzyme activity, leading to the enhanced growth performance and immune response observed in the *H. fossilis* fingerlings.

The immune response in fish exhibits a cascade of diverse reactions that aims to restore homeostasis in animals [50]. The transcription of complement component (C3), acute phase protein (transferrin) and a pro-inflammatory cytokine, i.e., IL-1β (interleukin 1-β) is generally considered a sign of enhanced immune response or immune stimulation [8, 30]. As a pro-inflammatory cytokine, IL-1β mediates a fast and vigorous response, inducing early inflammatory response in animals [103]. Transferrin is a multi-tasking globular protein that has vital physiological roles in antimicrobial activity, binding and transport of iron, as well as in differentiation, growth and cytoprotection processes [104]. The level of serum transferrin varies in response to stress conditions and infection, and as such is considered a potential biomarker for acute phase response in vertebrates [105]. In addition, complement system central component C3, is involved in both innate and adaptive immune response, and has several essential functions including direct killing of pathogens, opsonization, induction of immune response and inflammation regulation [106]. Research in fish has shown that exposure to microbial metabolic products and their derivatives results in an up-regulation of IL-1β, transferrin and C3 expression [107–109]. Significant up-regulation of transferrin, C3 and IL-1β gene transcriptions were observed in our study within microbial inoculum supplemented biofloc groups. The expression of transferrin and IL-1β genes were significantly enhanced at 35 days post treatment, while the maximum expression values of C3 gene was observed at 70 days post-treatment. The analysis of gene expression corroborated that microbial inoculum containing probiotics and immunostimulatory yeast products play an essential role in generating a protective immune response in *H. fossilis* fingerlings within the biofloc system.

Development of beneficial characteristics within supplemented biofloc systems appear to be dependent on the starter culture microbial composition. As such, careful selection of the composition of the microbial inoculum is critical to the development of an ideal biofloc system. Our study is a proof of concept for assessing microbial inoculum preparations as a biofloc developing agent for maintaining optimum water quality and bacterial diversity. The current trial also found that microbial inoculums improve gut histomorphology, growth performance and generates an antioxidant and protective immune response in *H. fossilis*. As our investigations have revealed the benefits of tailored microbial inoculums, further studies are recommended to explore the effects on other fish species, and expand the breadth of biofloc technology to enable widespread development of sustainable aquaculture.

## Supplementary Information

**Additional file 1. Fig. S1**: Activities during experiment period, **A** Fish stocking, **B**–**D** Sampling during the experiment period, and **E** flocs observation under the microscope. **Table S1**: List of primers used in the immune gene expression analysis of *H. fossilis*.

## Data Availability

The authors confirm that all relevant data are included in the article and/or its Supplementary information files.
