# Supplementary material for: Microbial inoculums improve growth and health of Heteropneustes fossilis via biofloc-driven aquaculture
Source: Microb Cell Fact. 2023 Jun 2;22:106. doi: 10.1186/s12934-023-02107-0 (PMC10239096; doi:10.1186/s12934-023-02107-0)
Supplement: Supplementary file 1 — Additional file 1. Fig. S1: Activities during experiment period, A Fish stocking, B–D Sampling during the experiment period, and E flocs observation under the microscope. Table S1: List of primers used in the immune gene expression analysis of H. fossilis. [file 12934_2023_2107_MOESM1_ESM.docx]

**Supporting information**

***
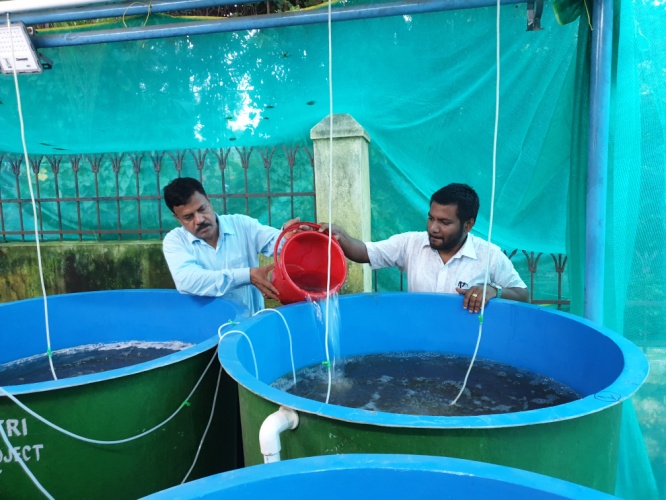

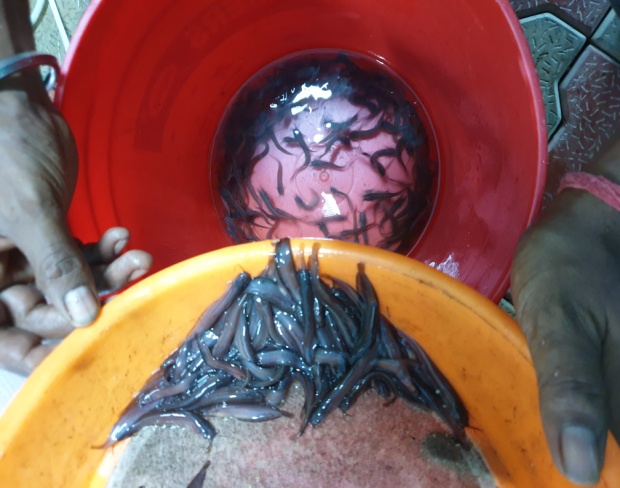

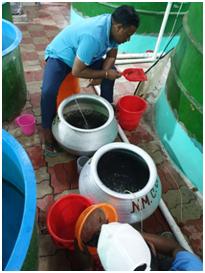

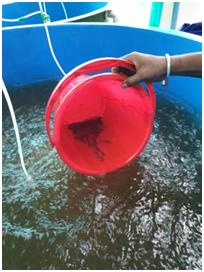

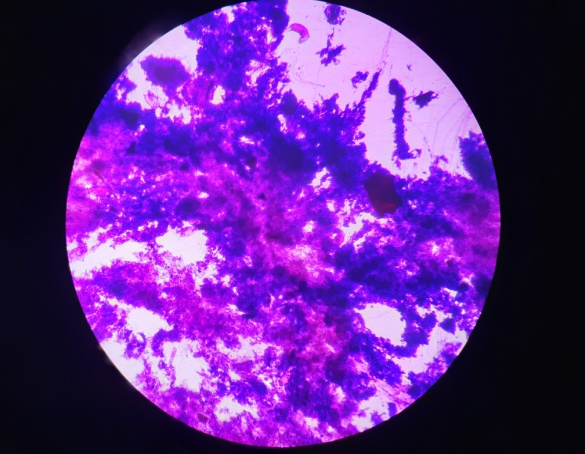
***

**C**

**D**

**E**

**B**

**A**

**Figure S1.** Activities during experiment period, (A) Fish stocking, (B-D) Sampling during the experiment period, and (E) Flocs observation under the microscope

**Table S1. List of primers used in the immune gene expression analysis of *H. fossilis***

| Gene name | Primer sequence (5′→3′) | Length (bp) | References |
| --- | --- | --- | --- |
| C3  (Complement) | FWD: TCCACCAGAGCCATCCCATA  REV: CACAACTTGAACGCCACCAG | 198 | Sirimanapong et al., 2015; Hoque et al., 2020 |
| Transferrin  (Acute phase response) | FWD: CACCCCATAACCTTCACCCC  REV: CGCAGTTTTCCCCAAACCAG | 149 |  |
| Interleukin-1β  (a pro-inflammatory cytokine) | FWD: CAGAGGCTGAAGCACACTCA  REV: CCTTGTCCTGCCTGCTGTAA | 148 |  |
| β -actin (Housekeeping reference gene) | FWD: ATTGATGCCCCTGGACACAG  REV: GGGTCTGTCCGTTCTTGGAG | 133 |  |
